# Overexpression of GRP78/BiP in P-Glycoprotein-Positive L1210 Cells is Responsible for Altered Response of Cells to Tunicamycin as a Stressor of the Endoplasmic Reticulum

**DOI:** 10.3390/cells9040890

**Published:** 2020-04-06

**Authors:** Mário Šereš, Lucia Pavlíková, Viera Boháčová, Tomáš Kyca, Ivana Borovská, Boris Lakatoš, Albert Breier, Zdena Sulová

**Affiliations:** 1 Institute of Molecular Physiology and Genetics, Centre of Biosciences, Slovak Academy of Sciences, Dúbravská cesta 9, 840 05 Bratislava, Slovakia; lucia.pavlikova@savba.sk (L.P.); viera.bohacova@savba.sk (V.B.); tomas.kyca@savba.sk (T.K.); ivana.sevcikova@savba.sk (I.B.); 2 Institute of Biochemistry and Microbiology, Faculty of Chemical and Food Technology, Slovak University of Technology in Bratislava, Radlinského 9, 81237 Bratislava, Slovakia; boris.lakatos@stuba.sk

**Keywords:** multidrug resistance, p-glycoprotein, tunicamycin-induced ER stress, GRP78/BiP, CHOP

## Abstract

P-glycoprotein (P-gp, ABCB1 member of the ABC (ATP-binding cassette) transporter family) localized in leukemia cell plasma membranes is known to reduce cell sensitivity to a large but well-defined group of chemicals known as P-gp substrates. However, we found previously that P-gp-positive sublines of L1210 murine leukemia cells (R and T) but not parental P-gp-negative parental cells (S) are resistant to the endoplasmic reticulum (ER) stressor tunicamycin (an N-glycosylation inhibitor). Here, we elucidated the mechanism of tunicamycin resistance in P-gp-positive cells. We found that tunicamycin at a sublethal concentration of 0.1 µM induced retention of the cells in the G1 phase of the cell cycle only in the P-gp negative variant of L1210 cells. P-gp-positive L1210 cell variants had higher expression of the ER stress chaperone GRP78/BiP compared to that of P-gp-negative cells, in which tunicamycin induced larger upregulation of CHOP (C/EBP homologous protein). Transfection of the sensitive P-gp-negative cells with plasmids containing GRP78/BiP antagonized tunicamycin-induced CHOP expression and reduced tunicamycin-induced arrest of cells in the G1 phase of the cell cycle. Taken together, these data suggest that the resistance of P-gp-positive cells to tunicamycin is due to increased levels of GRP78/BiP, which is overexpressed in both resistant variants of L1210 cells.

## 1. Introduction

The endoplasmic reticulum (ER) is a multifunctional membrane organelle with a complex interconnected structure that is involved in intracellular signal transduction [1] and protein synthesis, folding, modification, and quality control [2]. The processes of protein folding and maturation in the ER are controlled by accurate and strictly regulated mechanisms that depend on differential ER homeostasis, such as calcium storage/release equilibrium or equilibrium in pro-/antioxidant status. This homeostasis depends on the energy abundance/deprivation state of the cells and reflects metabolic stimulation, alterations in glycosylation, activation of inflammatory processes, and increases in protein synthesis. Misbalance in this complicated system leads to an increase in misfolded proteins in the cell [3], which is responsible for the development of ER stress in response to the accumulation of unfolded proteins (unfolded protein response (UPR)) within the ER lumen [4]. Initially, the UPR is a pro-survival mechanism that causes a reduction in the accumulation of unfolded proteins through depression of proteosynthesis and acceleration of proteosomal degradation [5]. However, if these mechanisms do not decrease the unfolded protein cell content, pro-death stimuli will be accelerated, and the UPR leads to the induction of programmed cell death. The UPR is mediated through three ER transmembrane receptors: pancreatic ER kinase-like ER kinase (PERK), activating transcription factor 6 (ATF6), and inositol-requiring enzyme 1 (IRE1) [5]. Under normal cellular conditions, all three ER stress receptors exist in inactivated states via their linkage with the ER chaperone GRP78/BiP (glucose reacting protein 78, also known as binding immunoglobulin protein), which is a regulator of the UPR [6]. When unfolded proteins accumulate within the ER lumen, GRP78/BiP dissociates from all three receptors and induces their activation, enabling them to trigger the UPR. Under these conditions, the UPR occurs via the following mechanisms: i. PERK specifically phosphorylates and inactivates the α-subunit of eIF2 (eukaryotic translation-initiation factor 2), leading to a rapid reduction in translational initiation and repression of global protein synthesis [7]. At a later time point, the PERK-ATF4 (ATF4: activating transcription factor 4, a cAMP-response element binding protein) pathway induces CHOP expression [8]. CHOP is known as C/EBP homologous protein (product of DNA damage-inducible transcript 3 – DDIT3 gene) and is a proapoptotic transcription factor [9]; ii. ATF6 activates the transcription of endoplasmic reticulum-associated degradation (ERAD) proteins via the following cascade. Accumulation of misfolded proteins in the ER induces specific cleavage of ATF6 by Site-1 and Site-2 proteases [10]. The cytosolic portion of ATF6 is then translocated into nucleus where it induces transcription of ER chaperones (e.g., GRP78/BiP), protein degradation enzymes, and X-box binding protein 1 (XBP1) [11]; iii. IRE1α, which contains two functional enzymatic domains (endonuclease and trans-autophosphorylation kinase domains), homooligomerizes and autophosphorylates itself [12]. In this state, the phosphorylated oligomers exert an unconventional RNA splicing activity that removes an intron from X-box binding protein 1 (XBP1) mRNA, which results in translation of the spliced variant of XBP1 (XBP1s), a functional transcription factor. XBP1s upregulates ER chaperones and ERAD genes that facilitate recovery from ER stress [13].

Proteostasis, which is homeostasis of proteins by equilibrium between synthesis, folding, and proteosomal degradation, is controlled by highly conserved molecular chaperones that include GRP78/BiP and GRP94, calnexin, and calreticulin [14]. GRP94 is another glucose reactive protein and an ER chaperone with antiapoptotic activity [15]. Calnexin (CNX) and calreticulin (CRT), two Ca2+-dependent lectins/chaperones of the endoplasmic reticulum, play crucial roles in the retention of misfolded proteins in the ER and represent an important part of N-glycosylation-dependent protein quality control in the ER [16].

The process of N-glycosylation in the ER is effectively blocked by tunicamycin, an inhibitor of N-acetyl glucosamine-phosphate transfer from the respective UDP-amino sugar to dolichol phosphate [17]. This inhibitory action is responsible for tunicamycin-induced ER stress that leads to the UPR. In the penultimate decade of the last century, the possible therapeutic use of tunicamycin was studied. In this regard was shown that mice receiving inoculations of L1210 cells pretreated with 10 μM tunicamycin in vitro survived nearly twice as long as did mice receiving implants of untreated tumor cells [18]. However, in 1985 was noted that tunicamycin, by blocking the process of N-glycosylation of proteins in animal cells, induces a wide range of effects that result in its cytotoxicity and therefore has no therapeutic potential [19]. Although there are references in the current literature about the anticancer effects of tunicamycin on various types of neoplasia (e.g., colon cancer [20], breast cancer [21,22], and non-small cell lung cancer [23]), the therapeutic use of tunicamycin cannot be expected in the near future. Nevertheless, tunicamycin is a very useful tool for research oriented on ER stress, and as a specific inhibitor of ER protein folding, it is often used to induce stress in this organelle [24].

Leukemia cells, similar to other neoplastically transformed cells, have characteristically deregulated proliferation [25], with accelerated proteosynthesis to levels that may overburden the protein quality control capacity of the ER, eventually inducing proteotoxic ER stress [26]. Cell survival in these conditions strictly depends on the maintenance of proteostasis [27].

Leukemia cells treated with antileukemic drugs develop multidrug resistance (MDR), which is a phenomenon in which cells become resistant to a large number of chemicals with diverse structures and different mechanisms of antileukemic activity [28]. The predominant molecular cause of MDR is a massive increase in P-glycoprotein (P-gp) expression/drug efflux activity [29,30]. P-glycoprotein was first identified as a member of the ATP-binding cassette (ABC) transporter family, in which expression is associated with multidrug resistance in Chinese hamster ovary cells that were selected for resistance to colchicine [31]. P-gp is a 140-kDa polypeptide that is glycosylated to 170–180 kDa [32,33] and is localized predominantly in the plasma membrane in leukemia cells [34].

Variants of mouse lymphocytic leukemia L1210 cells that overexpress P-gp due to either selection with vincristine (R) or transfection with a human gene encoding P-gp (T) are considerably less sensitive to repeated culturing in media containing sublethal concentrations of tunicamycin than parental L1210 cells (S) [33]. Similar behavior was observed when P-gp-negative and P-gp-positive variants of human acute myeloid leukemia (AML) cells were compared [35]. Only the unglycosylated 140 kDa P-gp form was present in R and T cells after culturing in medium containing tunicamycin, but interestingly, this form of P-gp was either integrated into the plasma membrane or had full efflux activity as measured by a calcein retention assay [33]. We pointed out a possible link between P-gp-mediated MDR and changes in UPR and the subsequent cellular response to ER stress in a review [36].

In the present paper, we studied differences between P-gp-negative S and P-gp-positive R and T cell expression of several proteins known to be active in the response of cells to tunicamycin-induced ER stress. For this study, we used conditions (time of incubation and concentration of tunicamycin) at which the cell death effects were negligible, and most of cells did not undergo apoptosis.

## 2. Materials and Methods

### 2.1. Cells, Culture Conditions, and Transfections

The sensitive parental (P-gp-negative) mouse leukemic cell line L1210 (S) was obtained from the Leibniz-Institut DSMZ-Deutsche Sammlung von Mikroorganismen und Zellkulturen GmbH (Braunschweig, Germany) ACC-123. Two P-gp-positive variants of S cells were prepared either by selection with vincristine (R) [37] or by transfection of S cells with the human gene encoding full-length P-gp (T) [38]. Plasmid 10957 (pHaMDRwt), a retrovirus encoding the full-length P-gp cDNA [39], was obtained from Addgene (Watertown, MA, USA). All cell variants were cultured in RPMI 1640 medium containing 8% bovine fetal serum and 20 μg/L gentamycin (all from Gibco, Langley, OK, USA) in a humidified atmosphere with 5% CO2 at 37 °C. Cells were cultured in the absence or presence of 0.1 μM tunicamycin (Sigma Aldrich, St. Louis, MO, USA) for different time periods (4, 8, and 24 h) prior to the experiment.

To increase GRP78/BiP levels in S cells, transient transfections with the pcDNA3.1(+)-GRP78/BiP plasmid (32701, 1 μg) [40] and with the control plasmid pCMV-PL (20783, 1 μg) [41], both from Addgene, using Lipofectamine 2000 reagent (Thermo Fisher Scientific, Bremen, Germany) were performed according to the manufacturer’s protocol.

### 2.2. Effect of Culturing in Media Containing Tunicamycin on the Viability of S, R and T Cells

The cells (S, R, and T; inoculums: 1 × 10^6^ cells) were cultured in 4 mL RPMI 1640 media with L-glutamine (1 μg/mL), 8% fetal bovine serum, and 1 μg/mL gentamycin (all purchased from Gibco, Langley, OK, USA) in a humidified atmosphere with 5% CO2 at 37 °C for 48 h in the absence or presence of tunicamycin (0.1 μM). This procedure was termed passaging and was repeated three times. The number of viable cells after each passage was counted using a CASY Model TT Cell Counter (Roche Applied Sciences, Madison, WI, USA). R cells were cultured for two passages without vincristine (VCR) prior to the experiments.

### 2.3. Detection of Tunicamycin-Induced Apoptosis and Necrosis in S, R, and T Cells

Cells (1 × 10^6^ cells per mL) were incubated for 4, 8, and 24 h in the presence of 0.1 μM tunicamycin under standard culture conditions. The proportions of apoptotic and necrotic cells were then detected using a fluorescein isothiocyanate -labeled annexin V (FAV) and propidium iodide (PI) kit (Calbiochem, San Diego, CA, USA). According to the procedure described by the manufacturer, the cells were washed twice with Phosphate-buffered saline PBS and gently resuspended in binding buffer containing 0.5 μg/mL FAV. The mixture was then incubated in the dark for 15 min at room temperature and centrifuged (4000× *g*, 15 min). The resulting sediments were resuspended in binding buffer containing propidium iodide (final concentration of 0.6 μg/mL), and the samples were immediately counted by flow cytometry using an Accuri C6 flow cytometer (BD Bioscience, San Jose, CA, USA).

### 2.4. Monitoring cell cycle progression

Cells (10^6^ cells per mL) were cultured for 24 h with or without 0.1 μM tunicamycin under standard culture conditions, washed with PBS, and fixed for 60 min with ice-cold ethanol (70% *v*/*v*). Each sample was then treated for 30 min at 37 °C with RNase and stained with PI. Cell fluorescence was counted by flow cytometry on an Accuri C6. Doublet discrimination mode was used to exclude doublets by means of rating the PI fluorescence signal width (FL2-W) and area (FL2-A). S cells (untransfected and transfected with either control plasmid or plasmid encoding GRP78/BiP) were used in special set of experiments and were processed by same protocol as S, R, and T cell.

### 2.5. Western Blotting

The protein levels were semiquantitatively determined by Western blotting. Cells were harvested and lysed with SoluLyse reagent containing a protease inhibitor cocktail (both from Sigma-Aldrich, St. Louis, MO, USA) and centrifuged at 12,000× *g* for 10 min. Protein lysates (30 μg per lane) were separated by SDS–PAGE on a Mini-Protean gel electrophoresis system (Bio-Rad, Philadelphia, PA, USA). Proteins were transferred by electroblotting to a polyvinylidene fluoride membrane (GE Healthcare Europe GmbH, Vienna, Austria) and identified by using the following primary and secondary antibodies: rabbit polyclonal primary antibodies against GRP78/BiP, GRP94, IRE1α, ATF6, PERK, CHOP, Bcl-2, Bax, cyclin D1, CNX, and glyceraldehyde 3-phosphate dehydrogenase (GAPDH), all from Santa Cruz Biotechnology (Dallas, TX, USA); monoclonal primary antibodies against ATF4 and caspases 3 and 9 from Cell Signaling Technology, Inc. (Beverly, MA, USA); and goat antimouse/rabbit secondary antibody linked with horseradish peroxidase from Santa Cruz Biotechnology. The proteins were visualized with an enhanced chemiluminescence detection system (GE Healthcare Europe GmbH, Vienna, Austria) using an Amersham Imager 600 (GE Healthcare). Broad range protein molecular weight markers (Thermo Fisher Scientific, Bremen, Germany) were used for molecular weight estimations. The intensity of protein bands was quantified by densitometry by using Image Amersham™ image analysis software (GE Healthcare Europe GmbH, Vienna, Austria). All samples were analyzed in triplicate, and the intensity levels were normalized to GAPDH as a housekeeping protein. Significance was established using an unpaired Student’s *t*-test. Quantities of CHOP were also determined in S cells (untransfected and transfected with either control plasmid or plasmid encoding GRP78/BiP), which were processed by same protocol as S, R, and T cell.

### 2.6. Determination of ER Stress-Induced Factors and Chaperones by RT-PCR

Total mRNA was isolated from S, R, and T cells using TRI reagent^®^ (Sigma Aldrich, St. Louis, MO, USA) according to the manufacturer’s instructions. DNA removal was performed with DNase I (Thermo Fisher Scientific, Bremen, Germany), and reverse transcription of total RNA was performed by using a RevertAid™ H Minus First Strand cDNA Synthesis Kit (Thermo Fisher Scientific, Bremen, Germany) according to the manufacturer’s protocol. PCR was performed in a total volume of 25 μL using DreamTaq Green PCR Master Mix according to the manufacturer’s protocol (Thermo Fisher Scientific, Bremen, Germany). GAPDH expression was used as an internal standard. After heating at 94 °C for 3 min to inactivate the reverse transcriptase, the samples were subjected to 30 cycles of denaturation (95 °C, 45 s), annealing (58 °C for ATF6, PERK, IRE1α, CHOP, XBP1, GRP94, Bcl-2, Bax, cyclin D1, cyclin B, CNX and cyclin A; 57 °C for cyclin E; and 56.6 °C for GAPDH; 30 s) and extension (72 °C, 90 s), followed by a final extension at 72 °C for 10 min.

The PCR products were separated on a 1.7% agarose gel (Invitrogen, Life Technology, Bratislava, Slovakia) and visualized with GelRed™ nucleic acid gel stain (Thermo Fisher Scientific, Bremen, Germany) using an Amersham Imager 600 (GE Healthcare Europe GmbH, Vienna, Austria). The primer sequences used in this study are presented in Table 1.

### 2.7. Determination of Stress Response Proteins by qPCR

Total mRNA was isolated from L1210 cells using TRI reagent (MRC, USA) according to the manufacturer’s instructions. Reverse transcription was performed using the RevertAid™ H Minus First Strand cDNA Synthesis Kit (Thermo Fisher Scientific, Bremen, Germany) according to the manufacturer’s protocol. Primers and cDNA samples were mixed with iTaq Universal SYBR Green Supermix (Bio-Rad, Laboratories, USA) for qPCR. For the thermal cycle reactions, a CFX96 Real-Time System C1000 Touch Thermal Cycler (BioRad, Laboratories, USA) was used at 95 °C for 10 min and then 39 cycles at 95 °C for 10 s and at 55 °C for 30 s. The relative amount for each transcript was calculated by a standard curve of cycle thresholds for cDNA samples and normalized to the amount of β-actin. The polymerase chain reaction (PCR) was performed in triplicate for each sample, after which all experiments were repeated twice. The data were analyzed with Bio-Rad CFX96T software. Baseline levels for each gene were computed automatically. The results were quantified from Ct values according to the formula ΔΔCt = ΔCt sample–Δc housekeeping gene. Quantities of GRP78/BiP transcripts were also determined in S cells (untransfected and transfected with either control plasmid or plasmid encoding GRP78/BiP), which were processed by same protocol as S, R, and T cell.

### 2.8. Visualization of GRP78/BiP in S, R, and T Cells Using Immunofluorescence Confocal Microscopy

After culture, the cells were washed and resuspended in PBS and then transferred onto poly-L-lysine glass slides (Menzel-Glaser, Braunschweig, Germany). The bound cells were washed twice in PBS, fixed with cooled methanol (−20 °C) for 20 min, washed in PBS, and then blocked with 0.2% bovine serum albumin (BSA) in PBS for 1 h at 37 °C. The cells were then incubated with primary antibody against GRP78/BiP diluted 1:1000 for 1 h at 37 °C in PBS containing 0.2% BSA, washed twice in PBS containing 0.2% BSA, and then incubated with Alexa Fluor 488 donkey anti-goat antibody (Life Technologies Corporation, Eugene, OR, USA) in PBS containing 0.2% BSA for 1 h at 37 °C. The specimens were washed twice in PBS containing 0.2% BSA and were labeled with 10 mg/L 4’-6-diamidino-2-phenylindole (DAPI, Sigma Aldrich, St. Louis, MO, USA) in PBS to visualize the nuclei. Finally, the coverslips were mounted onto slides with a mounting medium (80% glycerol) and analyzed using a confocal laser scanning microscope (Nikon Eclipse Ti, Tokyo, Japan).

### 2.9. Detection of GRP78/Bip on Cell Surface of L1210 cells by Flow Cytometry

L1210 cells were cultivated in the absence or presence of tunicamycin (0.1 μM) for 24 h. Subsequently, the cells were washed three times with PBS, resuspended (5 × 10^5^ cells per mL) in RPMI medium with 5% of defatted BSA (Sigma Aldrich, St. Louis, MO, USA), and incubated for 30 min with anti-GRP78 antibody (described in previous chapter) in a humidified atmosphere supplemented with 5% CO_2_ at 37 °C. After incubation, the cells were washed three times with RPMI medium containing 5% BSA and subsequently left to interact with secondary antibody (Goat anti-Rabbit IgG linked with Alexa Fluor 660, A21074, Thermo Fisher Scientific, Bremen, Germany). The labeled cells were analyzed on BD Accuri C6 flow cytometer.

## 3. Results

### 3.1. Effect of Tunicamycin on S, R, and T Cell Cycle Progression

P-gp-positive R and T cells could be repeatedly cultured in medium containing tunicamycin at concentration 0.1 μM. In contrast, in S cells after the third passage, massive cell death was detected (Figure S1). However, a single passage in medium containing this concentration of tunicamycin did not induce massive cell death either in P-gp-negative S or P-gp-positive R and T cells (Figure S1 in Supplementary Materials); therefore, these condition was used in further experiments.

**Figure 1:**
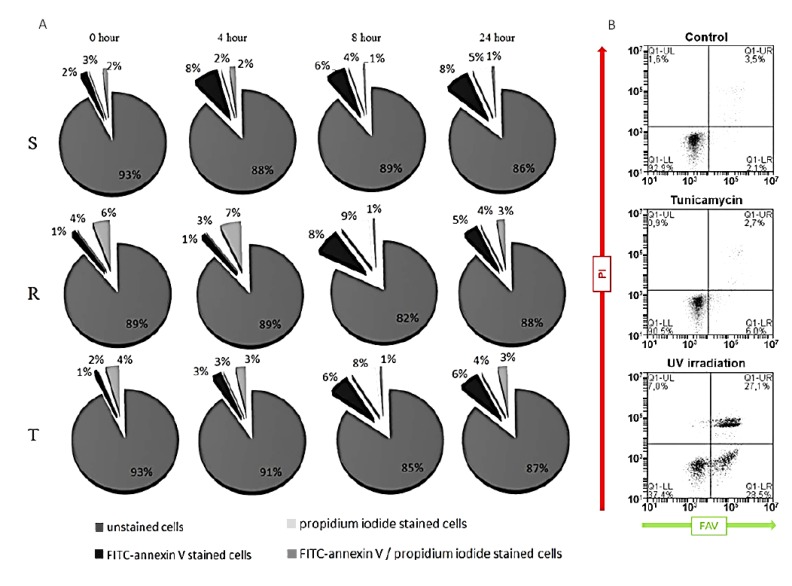
Determination of cell viability by fluorescein isothiocyanate -labeled annexin V (FAV)/propidium iodide (PI) assay: The cells were incubated for 24 h in the absence (control) or presence of 0.1 µM tunicamycin prior to the measurements. (**A**) Proportion of S, R, and T cells that were unstained (viable cells), stained with FAV (apoptotic cells), stained with PI (necrotic cells), and stained with both FAV and PI (late apoptotic/necrotic cells): Data in the pie charts are representative of three independent measurements. (**B**) Control apoptosis/necrosis induction after 10 min of UV irradiation of R cells using a germicide lamp and subsequent 24-h cell culture is shown as dot blots. Lower left quadrant: unstained cells; lower right quadrant: cells stained by FAV; upper left quadrant: cells stained by PI; and upper right quadrant: cells stained by both FAV and PI. Data are representative of three independent measurements, and similar data were also observed for S and T cells (not shown).

First, we focused on whether a single passage of S, R, and T cells at sublethal concentration of tunicamycin, which did not induce cell death, initiated apoptosis as measured by the FAV/PI kit. Incubation of S, R, and T cells in the presence of 0.1 μM tunicamycin for 4, 8, and 24 h did not induce any increase in the proportion of cells either in apoptosis (stained by FAV) or necrosis (stained with PI), and there were no cells in late apoptosis/necrosis that were stained for both of these markers (Figure 1A). To verify the ability of our experimental design to detect apoptosis, we exposed S, R, and T cells for 10 min to UV irradiation using a germicide lamp as a control for apoptosis induction [44]. Increases in the proportions of cells that were stained either by FAV, PI, or both of these markers were detected (as shown for R cells in Figure 1B).

**Figure 2:**
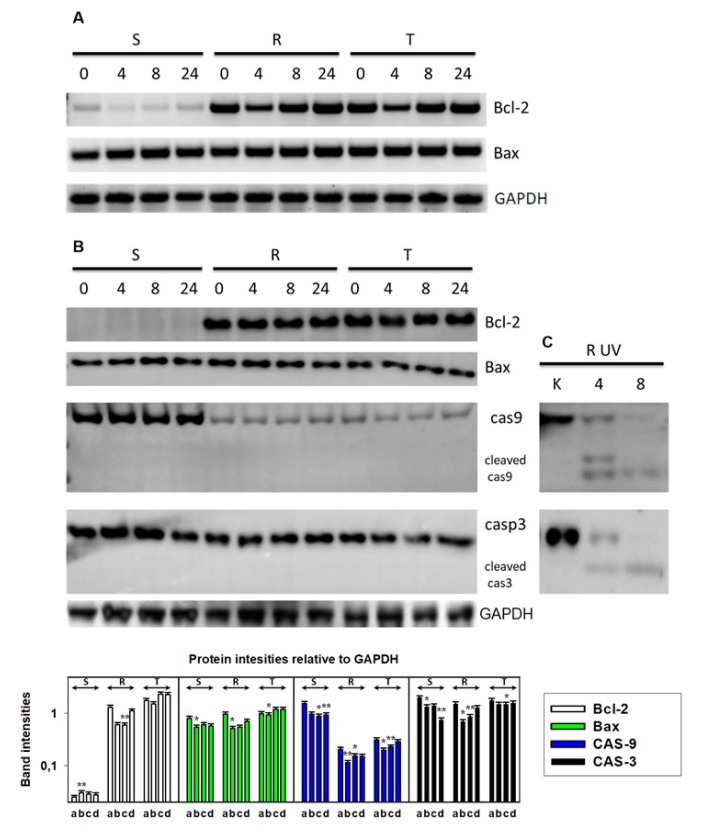
Expression of proteins that are active in apoptosis (Bcl2, Bax, and caspases 3 and 9 and their proteolytically activated forms) in S, R, and T cells after incubation in medium containing 0.1 µM tunicamycin for 0, 4, 8, and 24 h: (**A**) Detection of Bcl2 and Bax transcripts using RT-PCR and detection in agarose gel. (**B**) Western blot detection of Bcl2, Bax, and caspases 3 and 9: GAPDH was used as an internal control. Densitometry quantification of protein bands from Western blots (column plot expressed as the mean ± S.E.M. of three independent measurements of data relative to the GAPDH signal). Cells were incubated in the presence of 0.1 μM tunicamycin for 0 (a), 4 (b), 8 (c), and 24 (d). Significance: Data differ from values obtained in cells that were not incubated in the presence of tunicamycin (a) at the levels: * *p* < 0.02; ** *p* < 0.002. (**C**) Activated, proteolytically cleaved caspase 9 (upper) and caspase 3 (lower) as a control for caspase activation in R cells after 10 min of UV irradiation using a germicide lamp: After irradiation, the cells were incubated for 4 and 8 h in culture medium. Similar proteolytically cleaved forms of caspases after UV irradiation were also detected in S and T cells (not shown).

In the absence of tunicamycin, S cells expressed lower levels of the antiapoptotic Bcl-2 protein and almost identical levels of the proapoptotic Bax protein compared to those of R and T cells at both the mRNA and protein level (Figure 2A,B). A single passage of S, R, and T cells in the presence of tunicamycin did not considerably influence the expression of Bcl-2 and Bax at the mRNA or protein level (Figure 2A,B).

Increased levels of the initiating procaspase 9 protein and almost identical levels of the executioner procaspase 3 protein were detected by Western blotting in S cells compared with those in R and T cells (Figure 2B). However, culture of S, R, and T cells in the presence of tunicamycin did not induce alterations in the protein levels of either procaspase in S, R, and T cells; moreover, proteolytic cleavage to active caspases was not observed. In the control experiment, we demonstrated this proteolytic activation in S, R, and T cells after exposure to UV irradiation by a germicide lamp (as shown for R cells in Figure 2C). Thus, we may conclude that tunicamycin at a concentration of 0.1 μM does not induce cell death during a 24-h incubation period; therefore, we chose these conditions for subsequent experiments.

**Figure 3:**
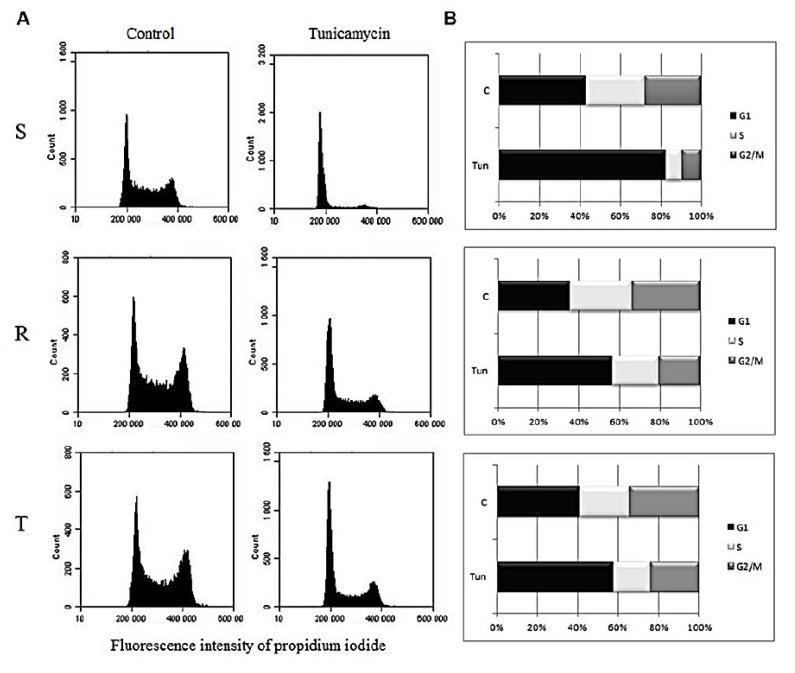
Effect of tunicamycin on the cell cycle of S, R, and T cells after 24-h incubation in culture conditions: (**A**) cell-cycle histograms of cells that were untreated C (control) and treated with tunicamycin for 24 h. (**B**) Summarization of cell cycle phases (G1, S, and G2/M) in column plots: Data are representative of three independent measurements.

Tunicamycin at a concentration of 0.1 μM induced an increase in the proportion of cells in the G1 phase of the cell cycle, which was associated with a decrease in the proportion of cells in the S and G2/M phases in S cells (Figure 3). However, in both P-gp-positive cells (R and T), retention of cells in the G1 phase was much less pronounced (Figure 3). 

**Figure 4:**
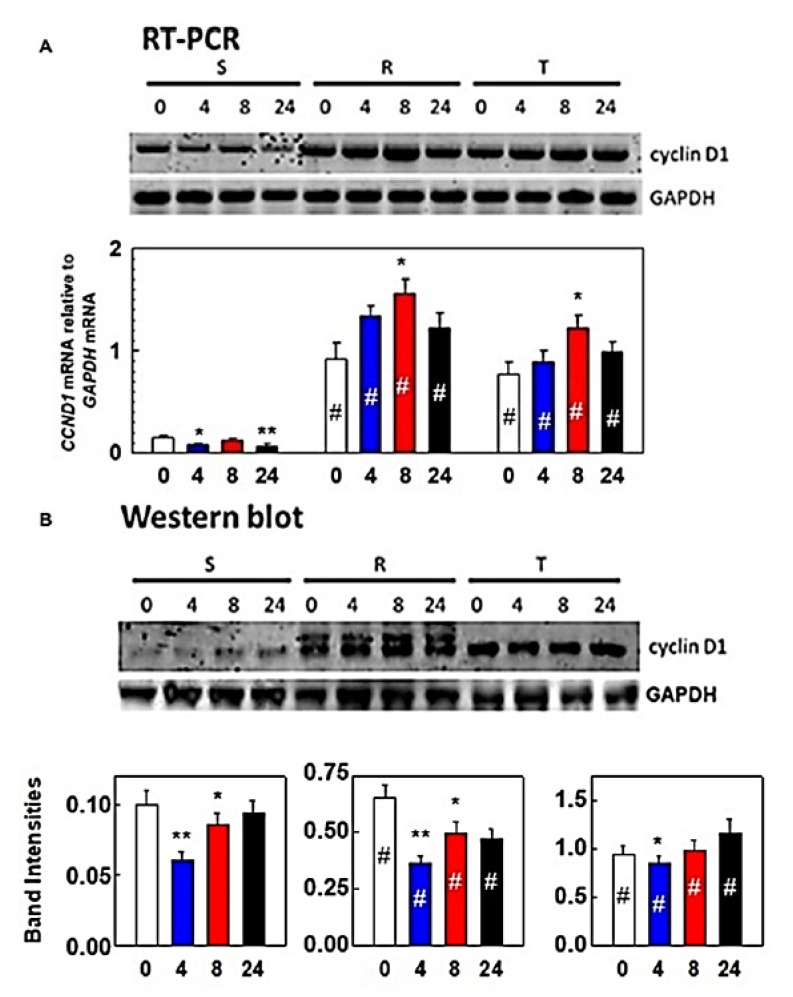
Expression of cyclin D1 in the presence or absence of tunicamycin: Respective mRNAs were detected by RT-PCR (**A**) and proteins were detected by Western blotting (**B**). S, R, and T cells were incubated in the presence or absence of tunicamycin for 4, 8, and 24 h. Signals for GAPDH mRNA and protein were used as internal controls. Data are representative of three independent measurements. Quantification of mRNA or protein signals obtained by densitometry are summarized in column plots. Data were normalized to GAPDH expression and are expressed as the mean ± S.E.M. of three independent measurements. Significance: Data differ from value obtained without incubation in the presence of tunicamycin (0) at the level * *p* < 0.05, ** *p* < 0.02; data differ from that correspondingly obtained for S cells at the level # *p* < 0.01. Expression of other cyclins A, B1, and E at mRNA levels are documented in the Supplementary Materials (Figure S2).

P-gp-negative cells (S) expressed lower levels of cyclin D1 than P-gp-positive R and T cells at both the mRNA and protein levels (Figure 4). Incubation of S, R, and T cells in medium containing tunicamycin at a concentration of 0.1 μM did not induce additional changes in cyclin expression at the mRNA or protein level.

### 3.2. Endoplasmic Stress Receptors and Activation of the UPR

**Figure 5:**
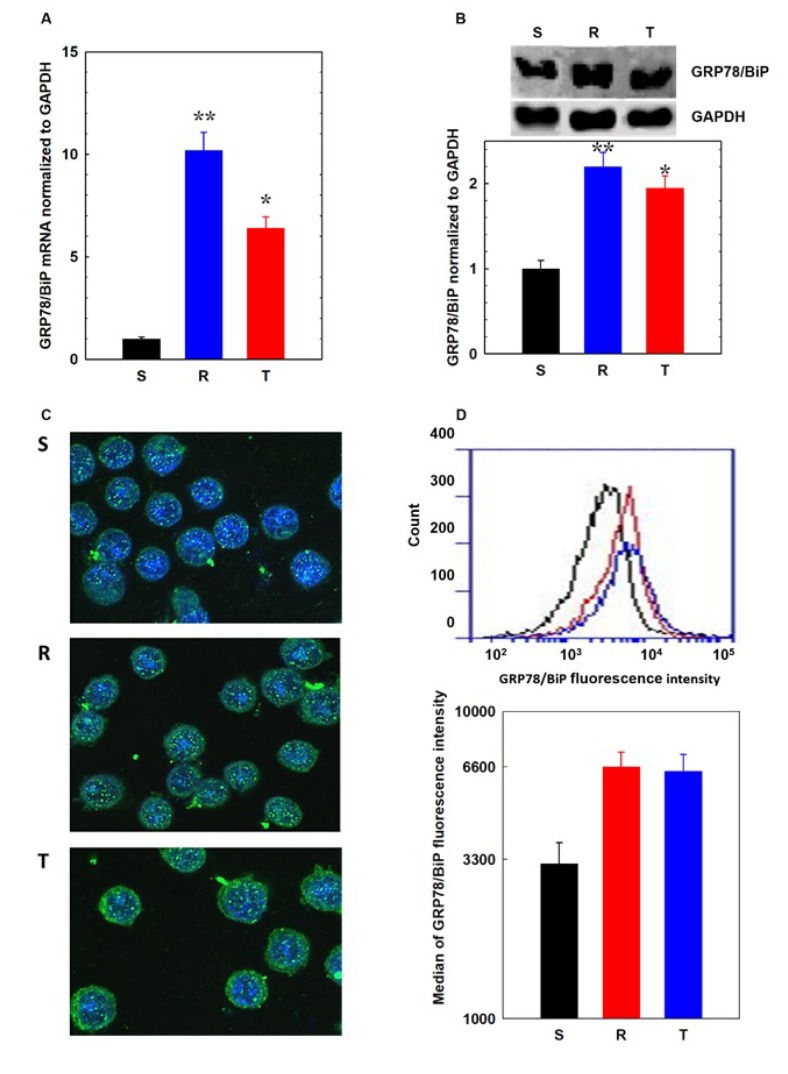
Detection of GRP78/BiP expression in S, R, and T cells: (**A**) Estimation of GRP78/BiP mRNA by qRT-PCR; data represent the mean ± S.E.M. of three experiments. Data differ from values obtained for S at the levels * *p* < 0.02.; ** *p* < 0.002. (**B**) Western blot detection of GRP78/BiP and its quantification: data are expressed as the mean ± S.E.M. of three independent experiments. Data differ from values obtained for S at the levels * *p* < 0.02.; ** *p* < 0.002. (**C**) Immunocytometric visualization of GRP78/BiP in S, R, and T cells using confocal microscopy of fixed and permeabilized cells (see Materials and Methods section); nuclei are stained by 4’-6-diamidino-2-phenylindole (DAPI) and are blue, and GRP78/BiP is stained with FITC-conjugated anti-GRP78/BiP antibody and is green; and data are representative of three independent measurements. (**D**) Detection of GRP78/BiP protein on the cell surface of living S, R, and T cells stained with FITC-conjugated anti-GRP78/BiP antibody using flow cytometry and their quantification in histogram medians in column plots: data are expressed as the mean ± S.E.M. of three independent experiments.

GRP78/BiP plays a crucial role in the ER stress response by inactivating endoplasmic reticulum receptors (PERK, IRE1, and ATF6) [6]. P-gp-negative cells contained less GRP78/BiP than P-gp-positive R and T cells at the mRNA (Figure 5A) and protein levels (Figure 5B). This protein was localized intracellularly and predominantly in structures around the nucleus (Figure 5C). Moreover, this protein was detected in R and T cells than in S cells. Some GRP78/BiP was also localized in the plasma membrane of cells, particularly in the R and T variants (Figure 5C). To quantify the cell surface GRP78/BiP, we performed flow cytometry counting of viable S, R, and T cells stained with anti-GRP78/BiP antibody (Figure 5D). These data showed slightly increased amounts of GRP78/BiP on the plasma membrane of R and T cells when compared with that of S cells. Incubation of S cells in tunicamycin-containing medium induced a strong increase in GRP78/BiP (Supplementary Materials Figure S3). Protein levels in cells that were incubated in the presence of tunicamycin for 24 h compared to cells without this treatment increased 10-fold. In P-gp (R and T)-positive cells in which higher levels of GRP78/BiP have been registered in the absence of tunicamycin, its increase is less pronounced and is about 2-fold.

**Figure 6:**
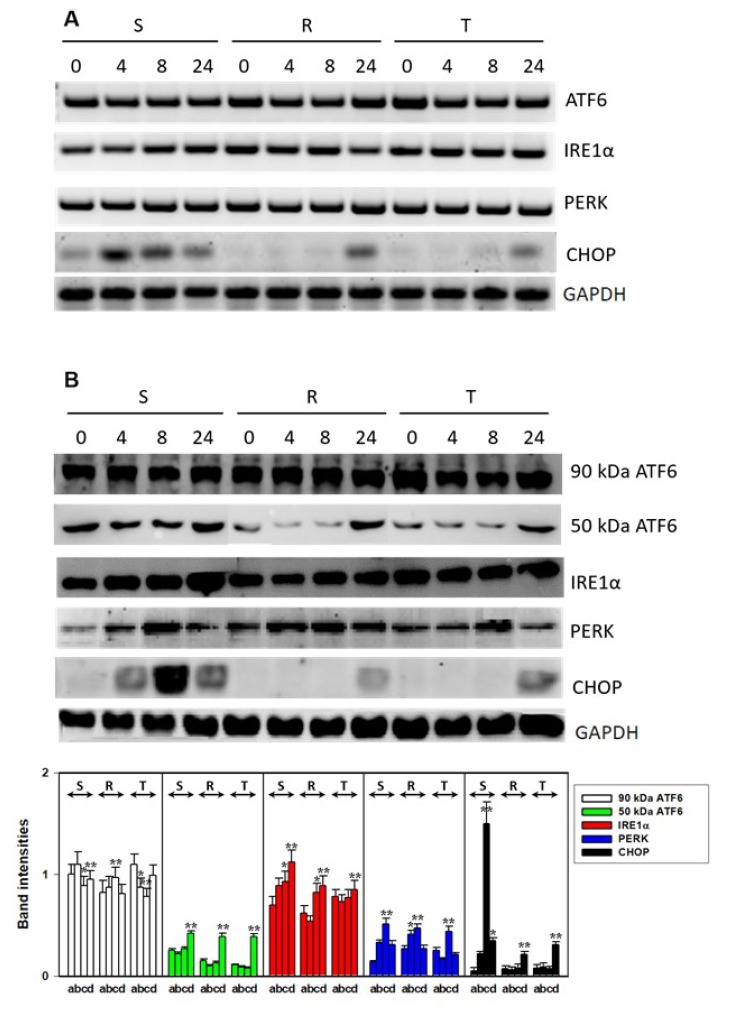
Detection of ER stress receptors/stress markers in S, R, and T cells after 4-, 8-, and 24-h incubation in the presence or absence of tunicamycin: (**A**) RT-PCR analysis of the ER stress-related genes ATF6, IRE1α, PERK, and CHOP. GAPDH expression was used as an internal control. Data are representative of three independent measurements. (**B**) Western blot analysis of the ER stress-related proteins ATF6 (and its cleaved fragment), IRE1α, and PERK and the ER-stress regulator/marker CHOP. GAPDH was used as a housekeeping protein. Protein bands were quantified by densitometry, and data in bottom column plots were normalized to GAPDH and are expressed as the mean ± S.E.M. of three independent measurements. Cells were incubated in the presence of 0.1 μM tunicamycin for 0 (a), 4 (b), 8 (c), and 24 (d). Significance: Data differ from values obtained in cells that were not incubated in the presence of tunicamycin (a) at the levels: * *p* < 0.02; ** *p* < 0.002.

Similar levels of mRNA encoding ATF6 and inactive 90 kDa ATF6 protein were found in S, R, and T cells, and these levels were unaffected by incubation of the cells in the presence of 0.1 μM tunicamycin (Figure 6A,B). However, the 50 kDa ATF6 fragment was present in both the drug-sensitive S and drug-resistant R and T cells, indicating the activation of the ATF6 receptor in all L1210 cell variants. This active ATF6 fragment was present in higher levels in S cells than in R and T cells (Figure 6B). Incubation of S cells in medium containing 0.1 μM tunicamycin did not induce any differences in the levels of the 50 kDa fragment. Increases in this fragment in R and T cells were similar to that of S cells only after incubation in the presence of tunicamycin for 24 h.

The PERK receptor mRNA was expressed in all variants of L1210 cells at equal amounts (Figure 6A). The mRNA level of this receptor was not affected by incubation in medium containing tunicamycin. In S, R, and T cells cultured with tunicamycin, the protein level of PERK increased slightly (Figure 6B). The downstream target gene of this receptor is the apoptotic transcription factor CHOP. The level of CHOP mRNA (Figure 6A) increased in sensitive cells cultured with 0.1 µM tunicamycin (namely after 4 h). In R and T cells, the increase in CHOP mRNA was detectable only after 24-h culture of cells in tunicamycin. Higher level of CHOP mRNA was detected in S cells than in R and T cells by qPCR (Figure 8). Moreover, strong increases in this transcript due to incubation in medium containing tunicamycin were detectable only in S cells. Increases in CHOP levels induced by tunicamycin were maximally detectable in S cells by Western blotting after 8 h of culture (Figure 6B). In resistant R and T cells, small but detectable amounts of CHOP protein were measured only after 24 h of culture in medium containing tunicamycin. 

**Figure 7:**
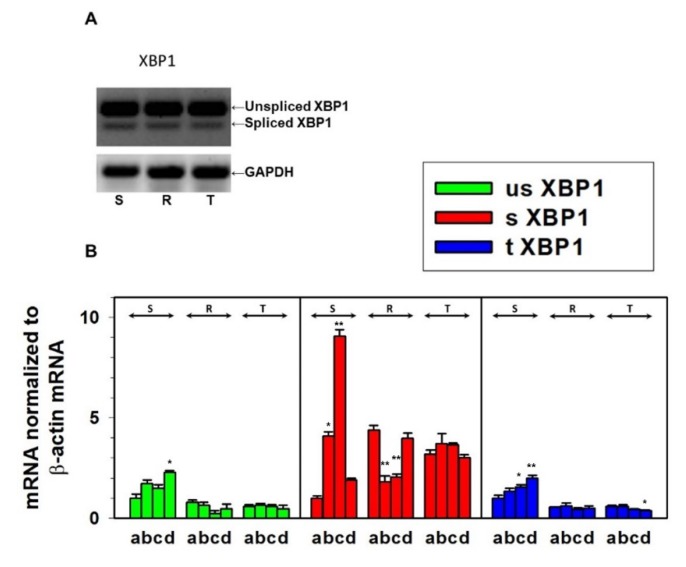
Detection of the unspliced and spliced variants XBP1 transcripts in S, R, and T cells: (**A**) RT-PCR identification of unspliced and spliced variants of XBP1 in S, R, and T cells in the absence of tunicamycin using primers listed in Table 1: Data are representative of three independent experiments. GAPDH was used as an internal control. (**B**) qRT-PCR quantification of total, spliced, and unspliced XBP1 transcripts (using primers listed in Table 2) in S, R, and T cells after 0 (a), 4 (b), 8 (c), and 24 (d) hours of incubation in the presence of tunicamycin: Transcript levels were normalized to GAPDH as a housekeeping gene and are expressed as the mean ± S.E.M. of three independent measurements. Transcriptions of all three forms (s, us, and t) obtained for untreated S cells was arbitrarily taken as one. Significance: Data differ from value obtained without incubation in the presence of tunicamycin (a) at the level * *p* < 0.02, ** *p* < 0.002.

The expression of IRE1 was present in all three variants of L1210 cells at the mRNA and protein levels (Figure 6). While in S cells incubated in medium with tunicamycin there was a slight increase in IRE1 expression at the mRNA and protein levels, such effects were not observed in R and T cells. IRE1 seemed to be activated because the product of its unconventional RNA splicing activity, spliced XBP1 mRNA [45], was present in all three cell variants (Figure 7A). Therefore, we used special qPCR primers for quantification of the unspliced (uXBP1), spliced (sXBP1), and total (tXBP1) variants of XBP1 mRNA (Table 2). While S cells contained higher amounts of uXBP1 and tXBP1 than R and T cells, both P-gp-positive variant L1210 cells (R and T) contained higher amounts of active sXBP1 in the absence of tunicamycin. In sensitive S cells, 0.1 μM tunicamycin induced an increase in the levels of all three XBP1 qPCR products to amounts exceeding the corresponding values observed for R and T cells. An exception to this effect for the sXBP1 variant occurred in S cells after 24 h of incubation in medium containing tunicamycin (Figure 7). The activated sXBP1 variant was maximally detected in S cells at 8 h of culture (Figure 7).

We also detected lower amounts of the endoplasmic reticulum Ca2+-dependent lectin/chaperone-CNX in R and T cells than in S cells at the protein level (Figure S5 in Supplementary Materials). However, at the mRNA level, we did not detect differences in CNX expression. GRP94 expression at the mRNA level did not differ in S, R, and T cells, and tunicamycin did not induce detectable changes (Figure S5 in Supplementary Materials). However, at the protein level, GRP94 was upregulated in all L1210 cell variants after 24 h of incubation in medium containing tunicamycin.

### 3.3. Downregulation of CHOP Expression After Transfection of S Cells With GRP78/BiP

**Figure 8:**
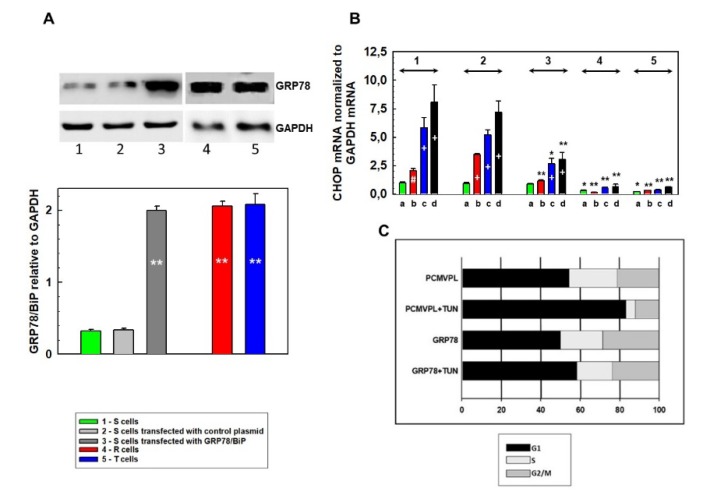
Effect of S cell transfection with the GRP78/BiP gene on CHOP mRNA transcription and cell cycle progression: (**A**) Western blot detection of GRP78/BiP in 1. S cells untransfected; 2. S cells transfected with pCMV-PL control plasmid; 3. S cells transfected with pcDNA3.1(+)-GRP78/BiP plasmid, 4. R cells, and 5. T cells. Data are representative of three independent measurements, and GAPDH was used as an internal control. Protein bands were quantified by densitometry, GRP78/BiP was normalized to GAPDH, and the data are expressed as the mean ± S.E.M. of three independent measurements in the column plot. Significance: Data differ from value obtained for untrasfected S cell at the level ** *p* < 0.002. (**B**) qRT-PCR quantification of CHOP transcripts in untransfected and transfected S cells, R cells, and T cells (symbols 1–5 are described above). Cells were incubated with tunicamycin for 0 (a), 4 (b), 8 (c), and 24 (d) hours. GAPDH was used as an internal control, and the data are expressed as the mean ± S.E.M. Transcription of *DDIT3* gene (encoding CHOP) obtained for untransfected S cells, which were untreated with tunicamycin, was arbitrarily taken as one. Data differ from value obtained without incubation in the presence of tunicamycin (a) at the level # *p* < 0.02, + *p* < 0.002; data differ from that correspondingly obtained for S cell transfected with control plasmid (2) at the levels * *p* < 0.02 and ** *p* < 0.01. (**C**) Proportion of S cells transfected either with pCMV-PL (PCMVPL) control plasmid or pcDNA3.1(+)-GRP78/BiP (GRP78) plasmid in different phases of the cell cycle (G1, S, and G2/M) after 24 h of incubation with 0.1 µM tunicamycin. Data are representative of three independent measurements.

The activity of endoplasmic receptors is negatively regulated through interactions with GRP78/BiP [6]. Increases in unfolded proteins within the ER lumen cause dissociation of GRP78/BiP from all three ER membrane receptors, which induces their activation. S cells that contain less GRP78/BiP respond to ER stressors more efficiently than R and T cells, which contain more of this protein (Figure 5). This may be responsible for arresting S cells in the G1 phase of the cell cycle after incubation with tunicamycin, which is much less pronounced in R and T cells (Figure 3). To explore this idea, we increased the intracellular level of GRP78/BiP in S cells by transfection with the pcDNA3.1(+)-GRP78/BiP plasmid (32701) as previously described [40]. The plasmid pCMV-PL (20783) was used as a negative control. Transfection of S cells with the plasmid encoding GRP78/BiP induced an increase in this protein to levels similar as in R and T cells, and approximately 6-fold increase was achieved compared to non-transfected or transfected control plasmid cells (Figure 8A). This increase in GRP78/BiP was associated with downregulation of *DDIT3* gene (encoding CHOP) transcription in GRP78/BiP-transfected S cells (compared with that of either untransfected cells or cells transfected with the control plasmid) that were cultured in the presence of tunicamycin (Figure 8B). Nevertheless, levels of this transcript still exceeded levels typical for R and T cells.

Similar to untransfected S cells (Figure 3), S cells transfected with the control plasmid and cultured in the presence of tunicamycin for 24 h induced the retention of cells in the G1 phase of the cell cycle (Figure 8C). However, in S cells transfected with GRP78/BiP, no similar retention in the G1 phase after culture in tunicamycin was detected (Figure 8C), and they responded to tunicamycin similarly to R and T cells (Figure 3). 

Therefore, we conclude that the tunicamycin-induced halt in the cell cycle at the G1 phase observed in S cells was causally linked to a lower level of GRP78/BiP, and the higher levels in R and T cells protected against tunicamycin-induced cell cycle arrest. Consistent with this finding, silencing GRP78/BiP induced cell cycle arrest in the G1 phase [46].

## 4. Discussion

Currently, we see an increased interest in the role of ER stress in the development of neoplastic cell resistance to therapeutics. Therefore, there is also an increased interest in the relationships between different MDR mechanisms and ER stress, resulting in their synergy in cellular mechanisms and ensuring reduced cell sensitivity to drugs. We recently published a review paper oriented on this topic [36]. The current paper is motivated by the effort to contribute to the understanding of the complex relationship in this issue. 

P-gp-positive variant L1210 cells (R and T) strongly expressed P-gp either at the mRNA or protein level compared with that of S cells [38]. Moreover, we used calcein retention assay to evaluate P-gp efflux activities and found that R and T cells possessed P-gp efflux activities but that S cells lacked this activity [33]. Both R and T cells were much less sensitive to P-gp substrates, such as VCR, doxorubicin, and mitoxantrone, than S cells [35]. All these features were periodically controlled in S, R, and T cells in our laboratory.

While R and T cells could be repeatedly cultured in medium containing sublethal concentrations of tunicamycin (0.1 μM), S cells did not endure these conditions well, and after the third passage, massive cell death was detected [33,35]. This result is also documented in the Supplementary Materials (Figure S1). However, a single passage in medium containing this concentration of tunicamycin did not induce massive cell death either in P-gp-negative S or P-gp-positive R and T cells (Figure S1 in Supplementary Materials). Consistent with our results, these conditions did not induce massive cell death in various cell models during a single passage, but when tunicamycin was applied at higher concentrations, cell death due to ER stress was detected [47,48,49].

In this study, we aimed to explain why P-gp-positive R and T cells are less sensitive to the ER stressor tunicamycin [33,35,50,51]. After passaging R and T cells with tunicamycin, P-gp protein was detected only in unglycosylated [33,51] and ubiquitinated [50] molecular forms, and this transporter was localized in its typical position in the plasma membrane with sustained calcein-efflux activity.

Another significant change in protein expression is the marked upregulation of Bcl-2 protein in R and T cells compared to S cells (Figure 2). The upregulation of Bcl-2 is often involved in MDR, and the concurrent upregulation of P-gp and Bcl-2 has been previously described in samples obtained from patients with AML and acute lymphoblasticleukemia (ALL) [52,53]. However, neither levels of anti-apoptotic Bcl-2 nor pro-apoptotic Bax protein were altered after culturing S, R, and T cells in the presence of tunicamycin (Figure 2). Reduced levels of procaspase 9 in R and T cells may reflect the antiapoptotic effect of upregulated Bcl-2 [54]. Both of these procaspases must be activated by specific proteolysis in response to the mitochondrial intrinsic apoptotic pathway [55]. However, we did not observe proteolytic activation of both caspases after a single culture of S, R, and T cells in the presence of tunicamycin. Therefore, it can be assumed that, under these conditions, there is no initiation of apoptosis processes, which was demonstrated also by double staining of cells with FAV and PI (Figure 1).

Although single culture of S cells does not result in apoptosis, these conditions induce cell retention in the G1 phase of the cell cycle. Similar behavior was not observed in R and T cells. The retention in the G1 phase of cell cycle in response to the presence of tunicamycin has been already described, and a time-dependent increase in the cell proportion in this phase in cells of hepatocellular carcinoma (Hep3B) after treatment with tunicamycin can be used as example [56]. However, the question remains why R and T cells are not retained in the G1 phase of the cell cycle after cultivation with tunicamycin. This may be caused (at least partly) by upregulation of cyclin D1 expression (on both mRNA and protein levels; Figure 4) in these P-gp positive cell variants. Cyclin D1 is known to be active in the transition of G1 to S phase of cell cycle [57] Expression of Cyclin E, another cyclin active in this cell cycle transition [58], was also found to be upregulated (at the mRNA level, Supplementary Material Figure S2) in R and T cells as compared to S cells. In contrast, the mRNA levels of cyclin A (which resides in the nucleus during S phase where it is involved in DNA replication [59]) and B1 (a G2/mitotic-specific cyclin [60]) were almost identical in S, R, and T cells, independent of the presence of tunicamycin (Figure S2 in Supplementary Materials). Therefore, differences in cell cycle regulation between P-gp-positive cells (R and T) and P-gp-negative S cells seem to originate from alterations in the G1/S phase transition. When tunicamycin was not present, the cell cycles of S, R, and T cells behaved similarly, but when tunicamycin was present, R and T cells continued in the cell cycle, in contrast to S cells, which preferentially halted in G1 phase (Figure 3).

By inhibiting the N-glycosylation process of proteins, tunicamycin blocks their folding in the ER and thereby induces accumulation of unfolded proteins. This causes ER stress and finally arrest of cell cycle in G1 phase [21]. GRP78/BiP is a central regulator of the cellular response to stress induced by accumulation of unfolded proteins in a cell [6]. Under these circumstances, GRP78/BiP dissociates from PERK, IRE1, and ATF6, which in turn mediate the response to ER stress. The fact that P-gp positive R and T cells have a higher cellular content of GRP78/BiP (Figure 4) than their P-gp negative counterpart S cells suggests that the former will be less stressed by tunicamycin-induced N-glycosylation retarding and subsequent accumulation of unfolded proteins. A small proportion of GRP78/BiP has been found on the cell surface in addition to its classical intracellular localization (Figure 4). Relocalization of GRP78/BiP from the ER to the plasma membrane was studied in 293T, HeLa, and MCF7 cells, and cell surface GRP78/BiP was assumed to play critical roles in cell signaling, proliferation, and overall survival [61].

**Figure 9:**
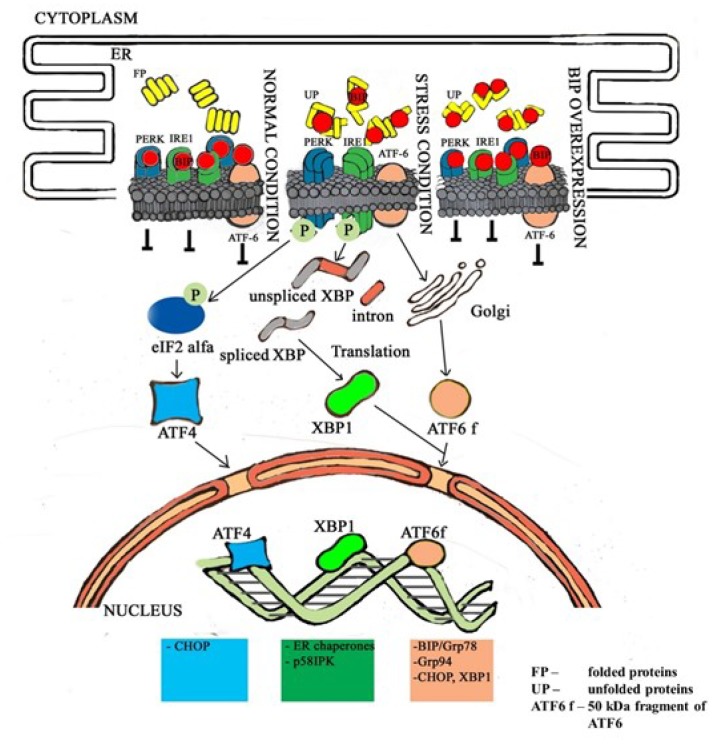
A schematic describing the altered response of R and T cells to tunicamycin-induced ER stress: In the absence of tunicamycin, all three ER stress receptors (PERK, IRE1, and ATF-6) are blocked by GRP78/BiP and therefore do not undergo downstream processes. In the presence of tunicamycin, when N-glycosylation of newly synthetized proteins is blocked, nonglycosylated/unfolded proteins accumulate in the ER and GRP78/BiP begins to interact with them. In S cells with lower GRP78/BiP levels, the amount of GRP78/BiP is insufficient to simultaneously bind unfolded proteins and to block all three ER receptors. Therefore, GRP78/BiP releases from PERK, IRE1, and ATF-6, and these receptors are activated and induce the following stress response pathways: i. PERK dimerizes, autophosphorylates, and subsequently inactivates eIF2α via specific phosphorylation. This leads to inhibition of eIF2 (alpha, beta, and gamma) heterotrimer formation. This results in a rapid decrease in translation or suppression of global protein synthesis. Due to this action, the arrest of S cells in the G1 phase of the cell cycle occurs. If this stress persists during repeated passages in the presence of tunicamycin, initiation of the PERK-ATF4 pathway induces CHOP expression; ii. IRE1 is homooligomerized, autophosphorylated, and exhibits RNA splicing activity that results in specific splicing of XBP1 mRNA and translation of the spliced variant of this protein. The truncated XBP1 variant is a functional transcription factor that initiates the expression of ER chaperones and endoplasmic reticulum-associated degradation (ERAD) genes. This allows cells to recover from ER stress. During this period, cell arrest occurs in the G1 phase; iii. after GRP78/BiP release, ATF6 is specifically cleaved and a 50-kDa fragment translocates into the nucleus, where it induces transcription of ER chaperones (e.g., GRP78/BiP), protein degradation enzymes, and XBP1. If the effect of sublethal concentrations of tunicamycin does not last long (during the first and second passages), the cells are able to grow normally in subsequent passages in the absence of tunicamycin. However, if this stress-induced condition persists, S cells do not proliferate, and after the third passage in the presence of tunicamycin, only a few viable cells were detected. R and T cells contain more GRP78/BiP, which is able to interact with larger amounts of unfolded proteins without release from the ER stress receptors. Under these circumstances, there is no activation of these receptors upon incubation with tunicamycin, as there is in S cells. Therefore, R and T cells are not retained in the G1 phase of the cell cycle under the influence of tunicamycin. Increasing GRP78/BiP in S cells by transfection with the gene encoding this protein prevented arrest in G1 cell cycle phase in transfected S cells after incubation with tunicamycin.

Upon release of GRP78/BiP from PERK, IRE1, and ATF6, these receptors activate and mediate their regulatory pathways within the UPR (Figure 9). Activation of PERK, IRE1, and ATF6 in all L1210 cell variants results from the presence of their downstream regulatory molecules: CHOP (Figure 6) ATF4 (Figure S4 in Supplementary Materials), spliced XBP1 (Figure 7), and the 50 kDa ATF6 fragment (Figure 6). The levels of CHOP, ATF4, 50 kDA ATF6, and both the spliced and unspliced forms of the XBP1 transcript are higher in P-gp negative S cells than in P-gp positive R and T cells. This indicates a higher level of ER stress induced by tunicamycin in S cells than in R and T cells. Interestingly, incubation of S cells in the presence of tunicamycin for 24 h leads to a decrease in the unspliced variant of the XBP1 transcript (Figure 7) and the protein level of CHOP (Figure 6). This downregulation is not observed for CHOP transcript (Figure 8).

CNX, a protein required for quality control of new proteins in ER, is less present in R and T cells than in S cells (Figure S5, Supplementary Material). We have previously described similar results comparing S and R cells [62]. Nevertheless, immunoprecipitation showed the existence of a complex of calnexin with P-glycoprotein in R cells, especially after incubation with VCR [63]. Since CNX together with another Ca^2+^-dependent ER lectin/chaperone calreticulin represents a crucial coupled player in protein quality control based on N-glycosylation in the ER [16], it is expected that P-gp-positive R and T cells differ from P-gp-negative cells in this process. Therefore, qualitative and quantitative differences in protein N-glycosylation could occur in L1210 cells when P-gp is overexpressed. Consistent with this, we previously detected differences between P-gp-negative S and P-gp-positive R and T cells in the specific binding of several lectins with cell surface-located sugars [35,38,50,51,64,65]. Culture of S, R, and T cells in the presence of 0.1 μM tunicamycin did not affect the expression of this CNX (Figure S5 in Supplementary Materials). The last protein that we detected in this set of experiments was GRP94, which is known to act as an antiapoptotic stimulus [15], and its expression on protein level seems to be elevated in S, R, and T cells after cultivation in medium containing tunicamycin (Figure S5 in Supplementary Material). 

In the case of CHOP, as the main inducer of apoptosis associated with ER stress [8], we observed its higher amount in S than in R and T cells. This appears to be responsible for a more pronounced response to tunicamycin in S than in R and T cells. This is consistent with the higher amount of GRP78/BiP in R and T cells than in S cells (Figure 5), since GRP78/BiP acts against the activation of PERK and the consequent induction of its downstream protein CHOP [8]. However, GRP78/BiP induces the elimination of CHOP by initiating its proteasomal cleavage because GRP78/BiP is known to interact directly with the N-terminal domain of CHOP, which accelerates CHOP ubiquitination and consequent degradation [66]. The latter possibility should eliminate CHOP protein without direct influence on transcription of the CHOP gene. However, the level of CHOP mRNA observed in S, R, and T cells in the absence or presence of tunicamycin seemed to be proportional to the level of CHOP protein (Figure 6). Thus, we propose that a higher level of GRP78/BiP induces changes in CHOP level between P-gp-positive R and T and P-gp-negative S cells that are likely due to a block in PERK-regulated transcription rather than through the initiation of CHOP proteasomal degradation. 

Taken all fact together, we may state that higher levels of GRP78/BiP in P-gp positive than in P-gp negative cells are responsible for sustained deactivation of all three ER receptor (PERK, IRE1, and ATF6), which finally caused the lack of cell cycle arrest in G1 phase in former cells treated by tunicamycin. In accordance with the latest statement are results with S cells transfected with a plasmid encoding GRP78/BiP, in which lower expression of CHOP was achieved, and upon tunicamycin treatment, lack of cell cycle arrest in the G1 phase was observed (Figure 8).

## 5. Conclusions

R and T cells with high P-gp expression grow during repeated passages in the presence of 0.1 µM tunicamycin without limitation [33,35,50]. P-gp-negative S cells do not have this ability. Consistently, we observed an increase in the proportion of S cells that were arrested in the G1 phase of the cell cycle after one passage in the presence of tunicamycin, which was not observed in R and T cells (Figure 3). In the present paper, we have shown that this ability results from changes in the expression of proteins that are involved in the cellular response to tunicamycin-induced accumulation of unfolded proteins during ER stress. The central factor is GRP78/BiP, which is upregulated in R and T cells (Figure 5) and induced more effective inhibition of the active forms of the three ER stress receptors PERK, ATF-6, and IRE1 (Figure 9). 

Tunicamycin induced the accumulation of nonglycosylated (unfolded) proteins to which GRP78/BiP bound upon its release from the ER stress receptors, which were subsequently activated. Cells with higher GRP78/BiP levels tolerate higher levels of unfolded proteins within the ER without activation of ATF-6, PERK, and IRE1 (Figure 9). Correspondingly, in R and T cells, we observed decreased levels of the downstream regulators CHOP and 50 kDa ATF-6 (after 4 and 8 h of incubation in the presence of tunicamycin), compared to those of S cells. To verify this finding, we transfected S cells with a plasmid to induce GRP78/BiP transcription/translation and downregulation of CHOP expression after incubation with tunicamycin was observed. Transfected S cells behaved similarly to R and T cells but differently from untransfected S cells in monitoring cell cycle changes under the influence of tunicamycin. Thus, we can conclude that an increased level of GRP78/BiP is responsible for the ability of R and T cells to survive repeated passage in media containing 0.1 µM tunicamycin.

## Figures and Tables

**Table 1:** Primers for RT-PCR.

Gene	Forward Primer	Revers Primer	bp
GAPDH ^b^	5′-TAT GTC GTG GAG TCT ACT GGT GTC-3′	5′-GTC ATC ATA CTT GGC AGG TTT CTC-3′	492
IRE1α ^b^	5′-AAC ACA CCG ACC ACC GTA TC-3′	5′-AGG GTC CTG GGT AAG GTC TC-3′	282
PERK ^b^	5′-GCC GAC GAT CAA ATG GAA GC-3′	5′-GTG GGG CTG AGG ATG GAA AA-3′	370
ATF6 ^b^	5′-TGG AAG TGG GAA GAT CGG GA-3′	5′-AGC CAC AGG TCC TCT TTA GG-3′	312
XBP1 ^a^	5′-GAA CCA GGA GTT AAG AAC ACG-3′	5′-AGG CAA CAG TGT CAG AGT CC-3′	us 205 s 179
CHOP ^b^	5′-GGA ACC TGA GGA GAG GTG TTC-3′	5′-TGC AGA TCC TCA TAC CAG G-3′	162
GRP94 ^b^	5′-GGG GAG GTC ACC TTC AAG TC-3′	5′-TGA GGG GGA GAT CAT CGG AA-3′	199
Cnx ^b^	5′-AGT GGG AAG TAG ATG AGA TGA AGG-3′	5′-ATA CAC CTG TCT TGG GAT TTT TGT-3′	333
cyclin D1 ^b^	5′-TCA CCC TGA GAG TAG GGA GC-3′	5′-GGC CTT CAG GCA AAA ACC AG-3′	592
cyclin A ^b^	5′-AGC AGA ACT CAT TCG GCT CT-3′	5′-CAA GGG AAA AGG AAG AAG AAG AGA A-3′	297
cyclin E ^b^	5′-ATG TTA CAG ATG GCG CTT GC-3′	5′-GAG GAC ACC ATA AGG AAA TCT GA-3′	254
cyclin B1 ^b^	5′-CAG TTG TGT GCC CAA GAA GA-3′	5′-CTA CGG AGG AAG TGC AGA GG-3′	216
Bcl-2 ^b^	5′-GGC TGG GGA TGA CTT CTC TC-3′	5′-GCA TGC TGG GGC CAT ATA GTT-3‘	323
Bax ^b^	5′-ATC CAA GAC CAG GGT GGC T-3′	5′-CTT CCC CCA TTC ATC CCA GG-3′	197

^a^ primers adopted from Reference [42]; ^b^ primers designed by program Primer 3 using the databases National Center for Biotechnology Information and Ensemblelibrary; us-unspliced XBP1 transcript; s-spliced XBP1 transcript.

**Table 2:** Primers for qRT PCR.

Gene	Forward Primer	Revers Primer
sXBP1 ^c,s^	5′-GTC CAT GGG AAG ATG TTC TGG-3′	5′-CTG AGT CCG AAT CAG GTG CAG-3′
usXBP1 ^c, us^	5′-GTC CAT GGG AAG ATG TTC TGG-3′	5′-CAG CAC TCA GAC TAT GTG CA-3′
totalXBP1 ^c,t^	5′-GTC CAT GGG AAG ATG TTC TGG-3′	5′-TGG CCG GGT CTG CTG AGT CCG-3′
CHOP ^c^	5′-AGG TGA AAG GCA GGG ACT CA-3′	5′-CCA CCA CAC CTG AAA GCA GAA-3′
GRP78/BiP ^c^	5′-TTT TCT GAT GTA TCC TCT TCA CCA GT-3′	5′-TTC AGC CAA TTA TCA GCA AAC TCT-3′
GRP94 ^c^	5′-CAA ATG GAG AAG ATT CCG CC-3′	5′-AAG AAT GAA GGA AAA ACA GGA CAA AA-3′
β-actin ^c^	5′-TGT CCA CCT TCC AGC AGA T-3′	5′-AGC TCA GTA ACA GTC CGC C-3′

^c^ primers adopted from Reference [43]; ^s^ primers for the spliced variant of XBP1; ^us^ primers for the unspliced variant of XBP1; ^t^ primers that detect both the spliced and unspliced variants of XBP1.

## Supplementary Materials

This paper contains Figures S1–S5 available online at https://www.mdpi.com/2073-4409/9/4/890/s1 in Supplementary Materials.

## Abbreviations

ABC	ATP-binding cassette
ALL	acute lymphoblasticleukemia
calcein AM	calcein acetoxymethyl
AML	Acute myeloid leukemia
ATF4	activatingtranscription factor 4
ATF6	activating transcription factor 6
ATF6/f	50-kDa proteolytic fragment of ATF6
Bax	Bcl-2-associated X protein
Bcl-2	B-cell lymphoma 2 protein
BSA	Bovine serum albumin
cAMP	Cyclic adenosine monophosphate
CDK4	Cyclindependentkinase 4
CHOP	C/EBP homologous protein
CNX	Calnexin
CRT	calreticulin
Ct value	cycle threshold value
DAPI	4′-6-diamidino-2-phenylindole
DMBA	dimethylbenzanthracene
DSMZ	German Collection of Microorganisms and Cell Cultures
eIF2	eukaryotic translation-initiation factor 2
ER	endoplasmic reticulum
ERAD	Endoplasmic-reticulum-associated protein degradation
FAV	Fluorescein linked annexin V
FP	folded protein
FITC	Fluorescein isothiocyanate
GAPDH	Glyceraldehyde 3-phosphate dehydrogenase
GRP78/BiP	glucose-regulated protein 78/Binding immunoglobulin protein
GRP94	glucose-regulated protein 94
SIRE1	inositol-requiring enzyme 1
MDR	multidrug resistance
MTT	3-(4,5-dimethyl-2-thiazolyl)-2, 5-diphenyl-2H-tetrazolium bromide
PBS	Phosphate-buffered saline
PCR	polymerasechain reaction
PERK	pancreatic ER kinase-like ER kinase
P-gp	P-glycoprotein
PI	propidium iodide
SDS-PAGE	sodium dodecyl sulfate–polyacrylamide gel electrophoresis
UPR	unfolded proteinresponse
UP	unfolded protein
UV	ultraviolet
s/us/t XBP1	spliced/unspliced/total X-boxbinding protein 1
qRT-PCR	quantitative RT-PCR
RPMI 1640	Roswell Park MemorialInstitute 1640 medium
HRP	horseradish peroxidase
VCR	vincristine
