# Supplementary material for: Overexpression of GRP78/BiP in P-Glycoprotein-Positive L1210 Cells is Responsible for Altered Response of Cells to Tunicamycin as a Stressor of the Endoplasmic Reticulum
Source: Cells. 2020 Apr 6;9(4):890. doi: 10.3390/cells9040890 (PMC7226765; doi:10.3390/cells9040890)
Supplement: Supplementary file 1 [file cells-09-00890-s001.pdf]

Supplementary Material

GRP78/BiP overexpression in P-glycoprotein-positive L1210 cells is responsible for resistance to tunicamycin-induced endoplasmic reticulum stress

Mário Šereš [1,*], Lucia Pavlíková [1], Viera Boháčová [1], Tomáš Kyca [1], Ivana Borovská [1], Boris Lakatoš [2], Albert Breier [1,2,*] and Zdena Sulová [1*]

[1] Institute of Molecular Physiology and Genetics, Centre of Biosciences, Slovak Academy of Sciences, Dúbravská cesta 9, 84505 Bratislava, Slovakia

[2] Institute of Biochemistry and Microbiology, Faculty of Chemical and Food Technology, Slovak University of Technology in Bratislava, Radlinského 9, 81237 Bratislava, Slovakia.

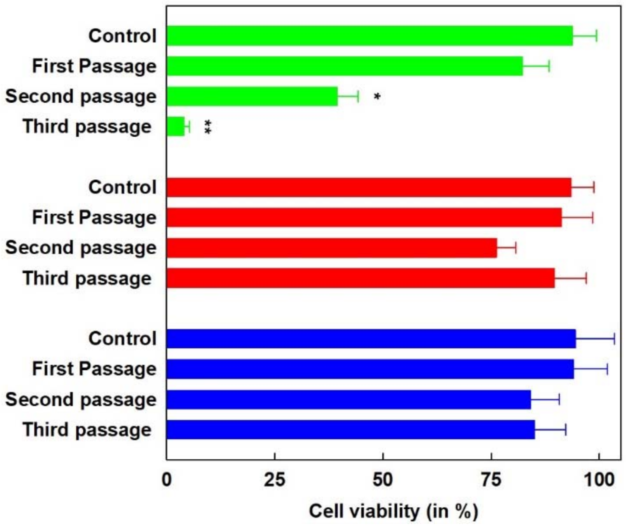

Figure S1. Cell viability of S, R and T cells after 1-3 passage in the presence of tunicamycin (0.1 μM) by using both propidium iodide and FITC-annexin V staining. The bars represent the mean ± S.E.M. (of three independent measurements) of the percentage of cells that were not stained with either propidium iodide or FITC-annexin V. S cells: green; R cells: red and T cells: blue. Significance: Data differ from control at the levels: *-P<0.02; **-p<0.01.

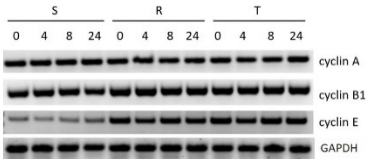

Figure S2. RT-PCR detection of cyclin A, B1 and E expression in S, R and T cells after 0, 4, 8 and 24 hours of incubation in medium containing 0.1 μM tunicamycin. GAPDH was used as an internal control. Data are representative of three independent measurements.

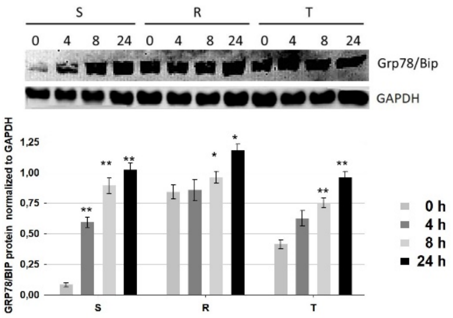

Figure S3. Levels of GRP78/BiP proteins in S, R and T cells after incubation of cells in medium containing 0.1 μM tunicamycin for 0, 4, 8 and 24 hours. Data are representative of three independent measurements. Protein bands were quantified by densitometry and data were expressed as means±S.E.M from three independent measurements. Significance: Data differ from values obtained in cells that were not incubated in the presence of tunicamycin at the levels: *- $p<0.02$; **- $p<0.01$.

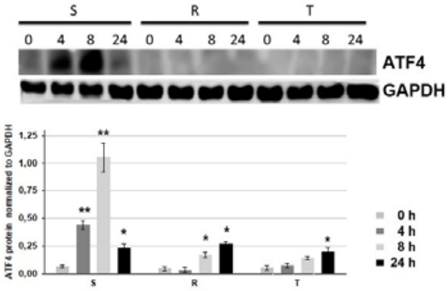

Figure S4. Levels of ATF4 proteins in S, R and T cells after incubation of cells in medium containing 0.1 μM tunicamycin for 0, 4, 8 and 24 hours. Data are representative of three independent measurements. Protein bands were quantified by densitometry and data were expressed as means±S.E.M from three independent measurements. Significance: Data differ from values obtained in cells that were not incubated in the presence of tunicamycin at the levels: *- $p<0.05$; **- $p<0.01$.

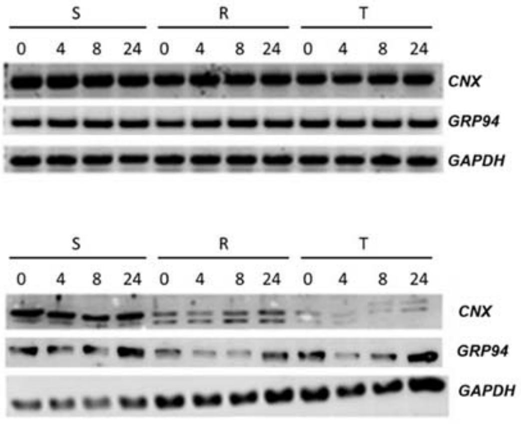

Figure S5. Expression of calnexin and GRP94 using RT-PCR (upper) and Western blotting (lower) in S, R and T cells after incubation of cells in medium containing 0.1 μM tunicamycin for 0, 4, 8 and 24 hours. Data are representative of three independent measurements.
